# Device Failures and Adverse Events Associated With Rhinolaryngoscopes: Analysis of the Manufacturer and User Facility Device Experience (MAUDE) Database

**DOI:** 10.2196/67036

**Published:** 2025-02-05

**Authors:** Shao-Hsuan Chang, Daishi Chen, Chi-Sheng Chen, Dong Zhou, Lung-Kun Yeh

**Affiliations:** 1 Department of Biomedical Engineering, Chang Gung University, Taoyuan, Taiwan; 2 Department of Otolaryngology, Shenzhen People’s Hospital, Shenzhen, China; 3 Department of Ophthalmology, Linkou Chang Gung Memorial Hospital, No. 5, Fuxing St, Guishan Dist, Taoyuan, 333423, Taiwan, 886 3-328-1200 ext 8666, 886 3-328-7998; 4 College of Medicine, Chang Gung University, Taoyuan, Taiwan

**Keywords:** medical device, device malfunction, rhinolaryngoscope, adverse event, MAUDE, Manufacturer and User Facility Device Experience

## Abstract

**Background:**

Rhinolaryngoscopes are one of the most widely used tools by otolaryngologists and speech-language pathologists in current clinical practice. However, there is limited data on adverse events associated with or caused by the use of rhinolaryngoscopes.

**Objective:**

In this study, we used the Manufacturer and User Facility Device Experience (MAUDE) database with the aim of providing insights that may assist otolaryngologists in better understanding the limitations of these devices and selecting appropriate procedures for their specific clinical setting.

**Methods:**

We characterized complications associated with the postmarket use of rhinolaryngoscope devices from the US Food and Drug Administration MAUDE database from 2016 through 2023.

**Results:**

A total of 2591 reports were identified, including 2534 device malfunctions, 56 injuries, and 1 death, from 2016 through 2023. The most common device problem with rhinolaryngoscopes was breakage (n=1058 reports, 40.8%), followed by fluid leaks (n=632 reports, 24.4%). The third most common problem was poor image quality (n=467 reports, 18%). Other device issues included contamination or device reprocessing problems (n=127 reports, 4.9%), material deformation or wear (n=125 reports, 4.8%), and device detachment (n=73 reports, 2.8%). Of the 63 reported adverse events, the most common patient-related adverse event was hemorrhage or bleeding, accounting for 18 reports, with the root causes including material deformation or wear, breakage, wrinkled rubber, or improper operation.

**Conclusions:**

Our study offers valuable insights for endoscopists and manufacturers to recognize potential issues and adverse events associated with the use of rhinolaryngoscopes. It emphasizes the need for improving device reliability, training, and procedural protocols to enhance patient safety during diagnostic procedures.

## Introduction

Rhinolaryngoscopes are currently available in various forms, including rigid, flexible, and video versions. They are essential tools routinely used by otolaryngologists and speech-language pathologists [1,2]. Given the substantial proportion of patients who require a rhinolaryngoscopic procedure as part of a head and neck examination, rhinolaryngoscopy has become an indispensable diagnostic procedure in otolaryngological practice [3]. Modern rhinolaryngoscopes can have a distal diameter as small as 2 mm, are equipped with lighting, are flexible, and have photo and video capabilities, enabling direct visualization of the nose, throat, and airway in diverse clinical settings, including emergency, inpatient, and outpatient scenarios. The range of indications for rhinolaryngoscopy includes visualizing polyps, tumors, and sources of epistaxis in the nasal cavity and aiding in the identification of suspected tumors or adenoidal hypertrophy [4]. In the oropharynx or laryngopharynx, rhinolaryngoscopy can be instrumental in evaluating foreign bodies and potential airway obstructions from etiologies such as neoplasm and epiglottitis, obstructive sleep apnea, dysphagia, dysphonia, tonsillar hypertrophy, glossoptosis, laryngomalacia, and vocal fold lesions [5,6]. Rhinolaryngoscopy may also assist in assessing the severity of angioedema. An alternative visualization tool is the flexible fiber-optic laryngoscope [7]. While laryngoscopes may be less expensive and simpler to use than rhinolaryngoscopes, fiber-optic rhinolaryngoscopy provides clearer visualization and better access to the larynx anatomy. In general, imaging quality, including illumination, color fidelity, resolution, and accurate length representation, plays a pivotal role in visualizing abnormalities. Therefore, the choice of instrument is often determined by clinician preference or equipment capabilities [8].

Rhinolaryngoscopes are classified as a moderate-risk medical device and require a 510(k) submission for premarket review by the US Food and Drug Administration (FDA; regulation number 21 CFR 874.4760). Although rhinolaryngoscopy is considered a generally safe procedure with rare serious adverse events, complications such as mucosal tearing, damage to anatomic structures (particularly with rigid scopes), bleeding, and laryngospasm have been reported [9]. However, there is limited data regarding device failures for both rigid and flexible rhinolaryngoscopes, incidence rates, and adverse events associated with these devices. The FDA’s Manufacturer and User Facility Device Experience (MAUDE) database serves as a repository for adverse events and malfunction reports related to medical devices, and it has been used as a data source to study device-related adverse events [10,11]. In addition, capturing user experiences and integrating them into the design during medical device development has become an essential component for ensuring patient safety and device effectiveness. In this study, we used the MAUDE database to characterize postmarket complications associated with the use of rhinolaryngoscope devices from 2016 through 2023, with the aim of providing insights that may assist otolaryngologists in better understanding the limitations of these devices and selecting appropriate procedures for their specific clinical setting.

## Methods

### Search and Selection

MAUDE, a comprehensive postmarket surveillance database from the FDA, was chosen because of its extensive collection of medical device reports (MDRs) from both mandatory and voluntary reporters related to FDA-cleared medical devices [11]. Mandatory reporters comprise manufacturers or device user facilities, whereas voluntary reporters include physicians, patients, or other device consumers. The MDRs from MAUDE include device malfunctions and potential patient harms associated with device failures during procedures. Therefore, the event classifications used in this analysis include device malfunctions and adverse events among patients.

The MAUDE database was queried by searching for the product category “rhinolaryngoscope (Flexible or Rigid),” which included MDRs from January 2016 through December 2023. This search encompassed terms such as “rhino-laryngo videoscope,” “rhino-laryngo fiberscope,” “telescope,” and “single use endoscope.” Each MDR was then logged in a Microsoft Excel spreadsheet. Among the reports, items that may have been incorrectly classified, such as cystoscopes and arthroscopes, were manually removed. These misclassifications accounted for only a small portion of the reports and resulted from errors made by the reporters. The MDRs underwent manual review, including assessment of the date of the event, event type (device malfunction, injury, or death), and the root causes of the adverse event. Device failures were defined as reported instances of rhinolaryngoscopes not functioning as expected during a procedure.

### Data Extraction

The device failure problems were classified as (1) breakage, (2) fluid leakage, (3) poor image quality, (4) contamination, (5) material deformation, (6) device detachment, (7) unintended movement, (8) mechanical problems, (9) unidentified device or use problems, (10) device overheating, (11) improper or incorrect procedures, (12) moisture damage, (13) packing problems, and (14) electrical shorting. To minimize potential bias and ensure consistency in categorization, 2 reviewers (SHC and DC) independently screened and categorized the reported events. Adverse events among patients included all reported complications resulting in injury, harm, or death during procedures associated with rhinolaryngoscopes. Following the categorization of the failure problems and adverse events, statistical analysis was conducted. The frequency of each failure type and adverse event was calculated, and the results were expressed as the number and percentage of occurrences within each category.

Additionally, to assess the context of these device failures in clinical practice, we analyzed the US Medicare population using *Current Procedural Terminology* (*CPT*) codes associated with rhinolaryngoscope diagnostic procedures (excluding surgeries). Specifically, we selected *CPT* codes 31231 (nasal endoscopy, diagnostic; unilateral or bilateral), 31575 (laryngoscopy, flexible; diagnostic), and 92511 (nasopharyngoscopy with endoscope), which may correspond to diagnostic procedures involving rhinolaryngoscopes. The data were accessed under the “Research, Statistics, Data & Systems” section. The Centers for Medicare and Medicaid Services Part B National Summary Data File was used to obtain annual procedure data based on *CPT* codes 31231, 31575, and 92511. These codes were used to compare the number of procedure claims with the number of device failure reports, allowing us to explore the relationship between the frequency of rhinolaryngoscope use (as indicated by procedure billing) and the reported failure incidents. This comparison provide a picture of the relative incidence of device failures in clinical practice compared to the frequency of procedures conducted, shedding light on potential areas for improvement in device performance or clinical training.

### Ethical Considerations

This study involved querying the FDA MAUDE database and US *CPT* codes. These activities did not involve direct interaction with human subjects, nor did they involve the collection or analysis of personal health data. Therefore, such data queries typically do not require approval from an institutional review board. Publicly available databases like the FDA MAUDE and *CPT* codes generally do not raise ethical concerns. We analyzed publicly available data and did not collect personal data or perform clinical trials, so institutional review board approval was not required.

## Results

### Device-Related Problems

A total of 2591 reports were identified, including 2534 device malfunctions, 56 injuries, and 1 death among patients from 2016 through 2023. The number of reports submitted significantly increased in 2021, 2022, and 2023 (n=347, n=1147, and n=918 reports, respectively), constituting greater than 90% of all reports submitted during the study period. Device malfunctions and their incidence rates are presented in Table 1. The most common device problem with rhinolaryngoscopes was breakage (n=1058 reports, 40.8%), followed by fluid leakage (n=632 reports, 24.4%). The third most common problem was poor image quality (n=467 reports, 18%). Other device issues included contamination or device reprocessing (n=127 reports, 4.9%), material deformation or wear (n=125 reports, 4.8%), device detachment (n=73 reports, 2.8%), and unintended movement (n=55 reports, 2.2%), with the remaining issues each accounting for less than 1%. Mechanical problems, device overheating, packaging issues, and electrical shorting were relatively infrequent problems, as summarized in Table 2.

**Table 1.** Event types reported to the US Food and Drug Administration Manufacturer and User Facility Device Experience (MAUDE) database for rhinolaryngoscopes per year.

Year	Deaths (n=1), n (%)	Injuries (n=56), n (%)	Malfunctions (n=2534), n (%)
2023	0 (0)	14 (25)	918 (36.2)
2022	1 (100)	7 (12.5)	1147 (45.3)
2021	0 (0)	2 (3.6)	347 (13.7)
2020	0 (0)	10 (17.8)	37 (1.5)
2019	0 (0)	14 (25)	38 (1.5)
2018	0 (0)	2 (3.6)	21 (0.8)
2017	0 (0)	5 (8.9)	13 (0.5)
2016	0 (0)	2 (3.6)	13 (0.5)

**Table 2.** Device problems reported for rhinolaryngoscopes.

Device problems	Reports (n=2591), n (%)
Breakage	1058 (40.8)
Fluid leakage	632 (24.4)
Poor image quality; optical distortion	467 (18)
Contamination; device reprocessing problem	127 (4.9)
Material deformation; wear	125 (4.8)
Detachment of device	73 (2.8)
Unintended movement	55 (2.2)
Mechanical problem	12 (0.5)
Unidentified device or use problem	11 (0.4)
Overheating of device	10 (0.4)
Improper or incorrect procedure	6 (0.2)
Moisture damage	6 (0.2)
Packaging problem	5 (0.2)
Electrical shorting; flare or flash	4 (0.2)

We conducted a comprehensive analysis of device failures, examining variations across specific manufacturers, including Olympus, Storz, Pentax, and Gyrus. A high incidence of breakage and fluid leakage reports was associated with Pentax. In comparison, Olympus devices showed a breakage rate of 4.8% (50/1028). However, Olympus devices were also associated with the highest frequency of contamination reports, followed by Pentax. These higher failure rates could be linked to the relatively larger market share of these manufacturers. Additionally, we scrutinized the frequency of nasal endoscopies performed in the US Medicare population using *CPT* codes 31231, 31575, and 92511. In 2022, a total of 1,196,520 procedures were performed. Meanwhile, the MAUDE database reported 1155 device failures. This results in an estimated failure rate of approximately 1 per 1000 procedures.

### Patient-Related Adverse Events

Adverse events or complications associated with device problems were evaluated based on the MAUDE database. Adverse events in patients in reports included all instances of complications resulting in injury, harm, or death associated with the use of rhinolaryngoscopes. Adverse events in patients were categorized as (1) hemorrhage/bleeding, (2) a foreign body in the patient, (3) burns or thermal burns, (4) discomfort or pain, (5) anaphylactic shock, (6) edema, (7) laryngospasm and dyspnea, (8) unspecified tissue injury, (9) allergic reaction, and (10) death. The specified root cause of the adverse event included material and mechanical problems, device chemical contamination, improper operation, fluid leakage, overheating, and display issues, among others.

The adverse events in patients and their specified root causes are presented in Table 3. A total of 63 reported adverse events were identified, including hemorrhage/bleeding (n=18 reports, 28%), a foreign body in the patient (n=12 reports, 19%), discomfort/pain (n=10 reports, 16%), thermal burns (n=9 reports, 14%), anaphylactic shock (n=4 reports, 6%), edema (n=3 reports, 5%), laryngospasm and dyspnea (n=3 reports, 5%), unspecified tissue injury (n=2 reports, 3%), allergic reaction (n=1 report, 2%) and death (n=1 report, 2%). The most common patient-related adverse event was hemorrhage/bleeding, accounting for 18 reports, with the root causes including material deformation or wear, breakage, wrinkled rubber, or improper operation. One death was reported, with an unspecified cause. Material breakage, material wear, and improper operation were identified as the major root causes for the reported adverse events. In 2019, 1 case of edema was attributed to improper reprocessing and device contamination with chemicals. The manufacturer’s investigation determined that the customer had insufficiently wiped phthalaldehyde-based disinfectants off the endoscope during reprocessing, resulting in the possibility of anaphylactic shock. Table 4 displays the numbers of adverse events from 2016 through 2023, including thermal burns, foreign bodies in patients, and discomfort, reported in 2023.

**Table 3.** Adverse events in patients from rhinolaryngoscope device problems.

Adverse event	Root cause	Events (n=63), n (%)
Hemorrhage/bleeding	Material deformation or wear; breakage; rubber wrinkling; improper operation	18 (28)
Foreign body in patient	Detachment of device or device component; breakage; material fragmentation; wear; improper operation	12 (19)
Discomfort/pain	Material wear; breakage; material invagination; mechanical problems	10 (16)
Burns/thermal burns	Scratched material; cut or torn material; burst battery; device overheating; use of device problem	9 (14)
Anaphylactic shock	Material perforation; material cracks or holes	4 (6)
Edema	Unidentified device or use problem; allergic reaction due to improper reprocessing and device chemical contamination	3 (5)
Laryngospasm and dyspnea	Display problem/poor image; unspecified issue	3 (5)
Unspecified tissue injury	Fluid/blood leakage; material wear	2 (3)
Allergic reaction	Reprocessing agent	1 (2)
Death	Unspecified issue	1 (2)

**Table 4.** Number of adverse events based on year and type.

Events, n
	2023	2022	2021	2020	2019	2018	2017	2016
Hemorrhage/bleeding	0	0	0	2	12	0	3	1
Foreign body in patient	4	0	0	0	2	0	5	1
Discomfort/pain	5	2	0	1	2	0	0	0
Burns/thermal burns	3	1	1	1	0	1	1	1
Anaphylactic shock	0	0	0	4	0	0	0	0
Edema	0	0	0	0	1	0	2	0
Laryngospasm and dyspnea	0	0	0	0	3	0	0	0
Unspecified tissue injury	0	2	0	0	0	0	0	0
Allergic reaction	0	0	1	0	0	0	0	0
Death	0	1	0	0	0	0	0	0

## Discussion

### Principal Findings

This study examined adverse events associated with the use of rhinolaryngoscopes based on the MAUDE database, analyzing 2591 common device issues and 63 patient-related adverse events from January 2016 through June 2023. Breakage (40.83%) was the most common device issue, whereas hemorrhage (28.57%) was the most common patient-related adverse event, primarily attributed to material deformation or breakage. These findings underscore the overall low occurrence of adverse events associated with the use of rhinolaryngoscopes. Previous studies have highlighted mucosal tearing and bleeding as the most common complications, with laryngospasm occurring in less than 1% of procedures [12-14]. To prevent these complications, adequate nasal decongestion and limited force are recommended. However, our results show that improper operation or use problems frequently contributed as the root cause of adverse events, including hemorrhages, foreign bodies in patients, thermal burns, pain, and edema. Operator experience with the device may also influence the occurrence of adverse events. One customer reported a broken eyepiece on a rigid scope (report number 9610773-2023-03626). Following investigation, the manufacturer attributed the issue to wear and tear coupled with excessive force. Similarly, another report detailed a damaged lens at the distal end of a scope (report number 9610773-2023-02419). Upon examination, the manufacturer concluded that this damage stemmed from user error, improper handling, and the application of excessive force. Therefore, considerations of usability and ergonomics are imperative to enhance patient care [15-17].

Despite gathering 125 reports of material wear from the MAUDE database, which accounted for one-ninth of the total breakage reports, it is noteworthy that the majority of the reported failures were not attributed to routine wear and tear, as determined by the manufacturer’s investigation. This observation underscores the importance of scrutinizing reported failures to distinguish between typical wear-related issues and potentially more serious underlying concerns. It is evident that device failures may arise from various root causes beyond routine wear and tear, which manufacturers may not consistently highlight in their manuals. For instance, in the manual for the TJF-Q180V (Olympus) endoscope, there is an acknowledgment that repeated use and reprocessing of the endoscope and its accessories can lead to gradual wear and tear. This underscores the importance of thorough understanding of and adherence to manufacturer instructions, as well as recognizing potential factors contributing to device failures, beyond typical wear-related issues.

Moreover, it was observed that many cases of device failure could be linked to improper reprocessing practices. In this study, 127 device failures caused by rhinolaryngoscope contamination were reported. Although manufacturers provide recommendations for product use and reprocessing, Biadsee et al [18] reported that less than 20% of physicians adhere to the recommended decontamination process outlined by manufacturers. Another study, from Jiang et al [19], of rhinolaryngoscope device failure due to contamination using the MAUDE database from 2013 to 2019 revealed associations with laryngeal edema, rather than infection, highlighting 1 injury resulting from improper reprocessing procedures (insufficient disinfectant removal from the endoscope). Anaphylaxis resulting from the use of phthalaldehyde-based disinfectants has been reported in cases involving the cleaning of rhinolaryngoscopes and cystoscopes [20,21]. In this case (report number 9610877-2019-00238), the risk of injury from microbial infection may be less than the potential harm caused by the disinfectant solution itself. In response to this incident, 5 members of the otolaryngology department were identified and retrained as the personnel at the hospital did not properly reprocess the endoscope. Regarding the compatibility of disinfectants, the manufacturer of the endoscope recommends that facilities adhere strictly to the instructions provided by the disinfectant manufacturer, including specific parameters such as concentration, temperature, and exposure time. Furthermore, it is crucial to ensure thorough rinsing of internal channels, external endoscope surfaces, and components with clean water to remove any residual detergent solution, thereby minimizing the risk of adverse reactions or complications associated with disinfectant use. Our results identified 1 allergic adverse event associated with a reprocessing agent, and 3 cases of edema due to improper reprocessing or device use, confirming previous literature regarding rhinolaryngoscope contamination [22,23]. Compared to other endoscope types, such as bronchoscopes and duodenoscopes, the contamination rate of rhinolaryngoscopes is less commonly associated with patient harm or death [18]. This indicates a comparatively lower risk associated with rhinolaryngoscopes in contrast to other endoscopic examinations.

This study reviewed the MAUDE database and identified 9 cases of thermal burns in patients who underwent rhinolaryngoscope examinations over 8 years. Scope overheating was reported as a root cause, likely because of prolonged procedure times with the light source on or the brightness setting of the lamp. Several studies assessed the heat effects of endoscopes in otorhinolaryngology and recommended keeping light sources at the lowest effective intensity [24-26]. MacKeith et al [27] evaluated the amount of heat produced by endoscopes and showed that larger-diameter endoscopes attain a higher temperature. Chitnavis [28] demonstrated that even momentary proximity could cause a thermal burn to a patient’s skin, without generating smoke or fire. The risk of thermal injury may be associated with the light source, endoscope caliber, and angulation [25,27,29,30]. Consequently, available cameras are often equipped with a regulation system capable of automatic gain control in poor lighting situations [31].

Furthermore, it is noteworthy that the number of MDRs underwent a significant surge in 2021, 2022, and 2023, with 347, 1147, and 918 reports, respectively. This notable increase underscores the importance of continued vigilance and thorough investigation into the factors driving these trends to ensure the safety and efficacy of medical devices. The growth in frequency can be attributed to several major factors, including the increased prevalence of laryngeal diseases, heightened use of laryngoscopes in airway management, guideline recommendations, elevated demand for respiratory products, and the impact of the COVID-19 pandemic. However, there was no significant rise in the number of nasal endoscopies performed in the US Medicare population with *CPT* codes 31231, 31575, and 92511 from 2016 to 2022. Another plausible explanation for the increased MDRs could stem from new product launches and strategic activities by manufacturers, which have had a moderate impact on the laryngoscope market, influencing the growth in the frequency of MDRs.

The statistical analysis conducted using the FDA’s MAUDE database carries significant implications for clinical practice. These data not only help identify trends and patterns in device failures but also provide critical safety information for clinical health care. By analyzing the MAUDE data, we can identify failure types and frequencies associated with specific devices, such as rhinolaryngoscopes, thereby understanding their potential impact on patient safety. The data reveal the sources of device failures, including variations across manufacturers and models, which can aid clinical decision-makers in selecting devices based on more informed criteria. Furthermore, by examining the time trends of device failures in the MAUDE database, we can correlate these trends with medical policies or other external factors. This trend analysis can assist health care institutions in adjusting operational processes and training programs to address the growing issue of device failures, especially during times of infectious disease outbreaks or when new devices are introduced. The data derived provide empirical evidence that can help in developing more effective clinical guidelines and training plans, ultimately contributing to improved quality of health care services [32]. These findings highlight the importance of timely adjustments to clinical operational processes and training programs to address the growing issue of device failures and provide guidance for future device improvements and clinical recommendations.

### Limitations

A major limitation of this study is its reliance on reports from the FDA’s MAUDE database, which may not account for comorbidities involved in reported device failures. Furthermore, the information may be incomplete or limited because MDRs can be submitted by health care professionals, patients, or manufacturers. Reporting variations between private-practice clinics and large academic medical centers may also affect data accuracy and which brands are used. Therefore, establishing causative associations for some reported adverse events is challenging. However, it is important to note that manufacturers are required to investigate reported adverse events and device failures, and their findings or follow-up reports are included in the MAUDE database. The manufacturer reports help to mitigate potential bias arising from self-reported data. Since the MAUDE database primarily focuses on device-related issues, we recognize the possibility of underreporting or inconsistent reporting practices. Nonetheless, the inclusion of both user-reported and manufacturer-investigated information adds a level of rigor to the analysis, allowing for a more nuanced understanding of the issues related to device failures. Despite these limitations, our study provides important insights for endoscopists and manufacturers to recognize potential issues and adverse events associated with the use of rhinolaryngoscopes, emphasizing the importance of patient safety.

### Conclusions

Although our findings underscore the overall low occurrence of adverse events associated with the use of rhinolaryngoscopes, the results indicate that improper operation frequently contributed to adverse events, including hemorrhage, foreign bodies in patients, thermal burns, pain, and edema. Operator experience with the device may also influence the occurrence of adverse events. Results from this study will be important for endoscopists and manufacturers to have a thorough understanding of the equipment and its limitations. Future research should assess the broader organizational impact, including otolaryngology teams, documentation practices, clinician training, and patient perspectives.

## Abbreviations

*CPT*: *Current Procedural Terminology*
FDA: Food and Drug Administration
MAUDE: Manufacturer and User Facility Device Experience
MDR: medical device report
